# An investigation into non-suicidal self-injury and its influencing factors among middle school students in a city in Hebei province

**DOI:** 10.3389/fpubh.2025.1564746

**Published:** 2025-07-17

**Authors:** Yanan Liu, Na Li, Yafan Li, Meng Wang, Yongliang Wang, Yanan Chen, Ling Hang, Jianan Ling, Wenting Lu, Minglong Gao

**Affiliations:** Department of Psychiatry, The First Hospital of Hebei Medical University, Shijiazhuang, Hebei, China

**Keywords:** non-suicidal self-injury, middle school student, influenced factor, investigation, China

## Abstract

**Objective:**

To investigate the incidence and influencing factors of non-suicidal self-injury (NSSI) among middle school students in a city within Hebei Province.

**Methods:**

A total of 11,321 students from grades one to three from two middle schools in Dingzhou, Hebei Province, were randomly selected. Socio-demographic data, mental disorders, coping styles, and experiences of childhood abuse were collected.

**Results:**

10,982 students finally included in the statistical analysis. We found that the detection rate of NSSI in the past year was 11.6%. Comparatively, the NSSI group and the non-NSSI group revealed significant discrepancies in coping styles, childhood abuse, and mental disorders. Factors protective against NSSI included living with parents, a problem-oriented coping style, and a preference for participating in extracurricular activities (OR = 0.729, 0.966, 0.815). NSSI's risk factors included frequent parental quarrels, interpersonal tension and sensitivity, depression, emotion-oriented coping styles, and childhood emotional abuse (OR = 1.322, 1.045, 1.141, 1.023, 1.137).

**Conclusion:**

The detection rate of NSSI within the past year in the city of Hebei stands at 11.6%. Considering the recognized risk and protective factors for NSSI, the possibility of NSSI may be decreased through the promotion of a harmonious home environment, encouraging positive emotional interaction, and the facilitation of greater involvement in extracurricular activities.

## Background

Non-suicidal self-injury (NSSI) refers to the deliberate, self-inflicted harm to one's own body without suicidal intent (1). Common forms of this behavior include scratching, beating, and burning oneself (2). NSSI is prevalently seen during adolescence, especially among female adolescents. Research indicates that the incidence of NSSI among international adolescents is 17.2% (3), while the detection rate among Chinese middle school students is 22.37% (4). Many studies have shown that NSSI occurs much earlier than attempted suicide and it is a strong predictor of attempted suicide or suicidal behavior (5, 6). The relationship between NSSI characteristics (such as cutting, burning, etc.) or high-frequency NSSI behaviors and suicide attempts is inseparable (6). These factors can significantly shorten the time from NSSI to attempted suicide. This finding is more obvious among girls and adolescents with depressive symptoms (5). The escalating issue of adolescent mental health problems is becoming a significant public health concern. NSSI not only directly endangers the physical health of teenagers and aggravates their emotional problems, but also may increase the risk of teenagers dropping out of school or even committing suicide (7). Recognizing NSSI behavior in adolescents and implementing intervention strategies for risk factors could potentially reduce the frequency of harmful outcomes, such as suicidal behaviors.

Prior research has demonstrated that NSSI in adolescents is associated with numerous factors. A troubled parent-child relationship is linked closely to NSSI. This relationship includes factors such as domestic violence or abuse, parental divorce or separation, limited parent-child contact, and a lack of family support, all of which are critical risk factors for NSSI (8, 9). Family factors play an important role in the early stage of teenagers' psychological development. Family also plays an important role in cultivating teenagers' social skills and emotional development, and these factors have a significant impact on NSSI (10). Xue et al. (9) found in their research that high family function is a strong protective factor for NSSI. Effective family communication can effectively prevent the occurrence of NSSI. On the contrary, dysfunctional families can increase the risk of NSSI by 21% (10). Additionally, strained interpersonal relationships, adverse childhood experiences, and symptoms of anxiety and depression can independently forecast the incidence of NSSI among adolescents (1, 2).

Wang et al. (11) discovered that adolescents who suffered childhood abuse were more likely to display NSSI behaviors compared to their healthier counterparts. Childhood abuse is an independent risk factor for NSSI in adolescents with a depressive disorder, with emotional abuse and emotional neglect being particularly impactful (1). Adverse childhood experiences can inflict short-term psychological and physical harm on individuals, and these effects can persist throughout their lifetimes (12). Prolonged adverse events during adolescence may trigger deviations in the developmental trajectory, impacting cognitive function development and personality trait formation (13), which subsequently increases the risk of NSSI in adolescents.

Coping style refers to the varying cognitive and behavioral efforts individuals employ to manage internal or external demands that exceed their capacities (14). Depending on their distinct functions and expression modes, coping styles can be categorized into problem-oriented and emotion-oriented styles. Prior research has indicated that emotion-oriented coping styles, typified by patience and evasion, can lead adolescents to choose damaging behaviors such as self-harm to evade reality (15). The act of self-harm might, in itself, represent a maladaptive coping style (16)—an improperly regulated method of dealing with negative emotions when more effective strategies are absent (17).

With the rise in detection rates of NSSI among adolescents, increased attention has been given by parents, medical professionals, and schools. However, there is a lack of large-sample screenings focusing on NSSI detection rates and influential factors among middle school students in Hebei Province. Consequently, this study aimed to estimate the NSSI detection rate among middle school students in Hebei Province via large-sample screening and to explore the impacts of mental disorders, coping styles, and childhood abuse on NSSI. The results of this study can provide a theoretical framework for preventing NSSI among adolescents.

## Methods

This study conducted a random selection of two middle schools in Dingzhou City, Hebei Province, and administered screenings to all students in these schools from May 2024 to June 2024. A total of 11,321 students were screened, but 52 of them declined to participate. We collected a total of 11,269 questionnaires, producing a recovery rate of 97.5%. After dismissing 287 invalid questionnaires, we included 10,982 valid questionnaires in the final statistical analysis.

The screening tool for this study is the information network evaluation system, designed by the Hebei Mental Health Technology Innovation Center. Students were required to complete the questionnaire by inputting their account number and password on the website. Upon logging in, students needed to provide informed consent before proceeding with the scale. If they disagreed, they would be considered to have withdrawn from participation in the screening.

In this study, a custom-made general information questionnaire was used to gather data on the subjects' gender, age, grade, parents' education level, whether they live with their parents, the frequency of their parents quarreling, and their regular participation in extracurricular activities.

### Ottawa self-injury inventory (OSI)

The OSI is employed to determine if the subject had engaged in NSSI within the past year, as well as the frequency, intent, location, and methods of NSSI implementation (18). This scale has commendable reliability and validity in evaluating the NSSI of adolescents (19). This study adopted the Chinese revised version of OSI, and the Cronbach's was 0.849.

### Coping style scale for middle school students (CSS)

CSS is used to assess how subjects typically respond to difficulties or challenges (20). It is divided into two subscales: one is the problem-oriented response subscale, and the other is the emotion-oriented response subscale. In this study, the Cronbach's α of this scale was 0.784.

### Mental health scale for middle school students (MSSMHS)

MSSMHS is used to gauge the comprehensive mental health level of middle school students (21). In this study, the Cronbach's α was 0.966.

### Childhood trauma questionnaire short form (CTQ-SF)

CTQ-SF, a self-assessment tool, is used to evaluate experiences of abuse (22). It is one of the most frequently utilized instruments to measure childhood abuse and neglect. This scale has commendable reliability and validity in evaluating the childhood abuse of adolescents (23). In this study, the Cronbach's α was 0.717.

The investigators included staff from the Mental Health Center of the First Hospital of Hebei Medical University and high school teachers from the schools involved in the screening. The investigators received uniform training before the screening, with uniform guidelines in place to ensure consistency in the screening results. During the screening process, students were instructed to think independently and fill out the Symptom Self-Assessment Scale according to the provided guidance. Upon completion, the students clicked the submit button. After the screening, the completed scales submitted by the students were reviewed, and questionnaires with obvious errors or incomplete answers were discarded.

The statistical analysis was conducted using SPSS 25.0 software. The Chi-square test was employed for the comparison of categorized data, while the two-independent sample *t*-test was applied for the comparison of continuous variables. To analyze the influencing factors of NSSI, logistic regression analysis was utilized, with a *P* value of < 0.05 indicating statistical significance.

This study received approval from the Clinical Research Ethics Committee at the First Hospital of Hebei Medical University [Approval number: (2024) Research Review No. (091)].The authors confirm that all research was performed in accordance with relevant guidelines/regulations.

**Consent statement:** Verbal and written consent was obtained from adolescent and parents/caregivers.

## Results

A total of 11,321 middle school students were screened in this study. Among them, 52 did not sign the informed consent form and did not participate in the screening. 287 questionnaires with incomplete data were excluded, and finally 10,982 people were included in the statistical analysis. According to OSI, if one subject showed any of the self-harm patterns once or more, it is determined to have NSSI behavior (24). Students screened out with NSSI behaviors were classified as the NSSI group (*n* = 1,278), and the remaining students were classified as the non-NSSI group (*n* = 9,704). It was found that the detection rate of NSSI in the past year was 11.6%. The average age of the NSSI group was 16.14 years old, and that of the non-NSSI group was 15.50 years old. Among the adolescents with NSSI, there were 745 girls and 533 boys. The number of girls was significantly higher than that of boys (χ^2^ = 34.164, *P* < 0.001) (Table 1).

**Table 1:** Comparison of general data of NSSI patients and non-NSSI patients.

**Category**	**NSSI group (*n* = 1,278)**	**Non-NSSI group (*n* = 9,704)**	***x*^2^/*t* value**	***P*-value**
Age (years)	16.14 ± 1.12	15.50 ± 0.975	−1.137	0.256
**Gender**				
Boys	533 (41.7%)	4,891 (50.4%)	34.164	< 0.001
Girls	745 (58.3%)	4,813 (49.6%)		
**Grade**				
Grade 1 senior	457 (35.8%)	3,741 (38.6%)	5.836	0.054
Grade 2 senior	453 (35.4%)	3,132 (32.3%)		
Grade 3 senior	368 (28.8%)	2,831 (38.6%)		
**Parental education**				
Both parents have lower education levels than high school	615 (48.1%)	4,625 (47.7%)	1.065	0.587
Father or mother has a lower education level than high school	425 (33.3%)	3,354 (34.6%)		
Both parents have higher education levels than high school	238 (18.6%)	1,725 (17.8%)		
**Live with parents**				
Yes	352 (27.5%)	1,739 (17.9%)	67.833	< 0.001
No	926 (72.5%)	7,965 (82.1%)		
**Parents often argue**				
Yes	344 (26.9%)	859 (8.9%)	377.816	< 0.001
No	934 (73.1%)	8,845 (91.1%)		
**Enjoy participating in extracurricular activities**				
Yes	625 (48.9%)	7,058 (72.7%)	305.097	< 0.001
No	653 (51.1%)	2,646 (27.3%)		

NSSI, non-suicidal self-injury.

Female adolescents primarily self-injured through scratches/abrasions and bites/pinches, while male adolescents' primary methods were scratches/bruises and blows, mainly focusing on arms, fingers/hands, and wrists. The main motivators behind NSSI were to release unbearable tension, discharge anger, alleviate sadness, and self-punishment. A statistical comparison revealed significant differences between the NSSI and the non-NSSI groups in terms of factors such as living with parents, frequency of parents' arguments, and affinity toward extracurricular activities (*P* < 0.001). However, there were no significant disparities regarding age, grade, and parental education levels between the two groups (Table 1).

As delineated in Table 2, notable differences were evident in coping styles, instances of childhood abuse, and prevalence of mental disorders between the two groups (*P* < 0.001). Students within the NSSI group, notably middle school students, appeared more inclined toward experiencing interpersonal difficulties, symptoms of anxiety and depression, increased academic stress, and a preference for emotionally charged coping mechanisms. These students in the NSSI group have been subjected to a greater number of adverse childhood experiences, including emotional neglect, emotional abuse, physical neglect, physical abuse, or sexual abuse.

**Table 2:** Comparison of MSSMHS, CSS and CTQ-SF between patients with and without NSSI.

**Category**	**NSSI group (*n* = 1,278)**	**Non-NSSI group (*n* = 9,704)**	***x*^2^/*t* value**	***P*-value**
**MSSMHS score**				
Obsessive-compulsive symptoms	13.33 ± 4.662	9.92 ± 3.652	−30.296	< 0.001
Paranoia	12.68 ± 5.288	8.54 ± 3.421	−37.712	< 0.001
Hostility	12.65 ± 5.656	8.11 ± 3.245	−40.249	< 0.001
Interpersonal sensitivity	14.09 ± 5.213	9.48 ± 3.889	−38.124	< 0.001
Depression	14.27 ± 5.413	9.05 ± 3.583	−45.663	< 0.001
Anxiety	15.11 ± 6.000	9.60 ± 4.189	−41.666	< 0.001
Learning pressure	14.64 ± 5.669	10.21 ± 4.440	−32.337	< 0.001
Maladjustment	12.62 ± 4.675	8.91 ± 3.311	−35.640	< 0.001
Mood swings	14.39 ± 4.849	10.07 ± 3.750	−37.318	< 0.001
Psychological imbalance	10.45 ± 4.403	7.99 ± 2.767	−27.585	< 0.001
**CSS score**				
Problem-oriented coping styles	49.30 ± 10.882	55.73 ± 11.339	19.744	< 0.001
Emotion-oriented coping styles	39.72 ± 9.185	34.28 ± 9.687	−19.772	< 0.001
**CTQ-SF score**				
Emotional abuse	7.46 ± 2.999	5.84 ± 1.500	−31.347	< 0.001
Emotional neglect	11.46 ± 4.718	9.75 ± 4.345	−13.090	< 0.001
Physical abuse	5.74 ± 1.978	5.19 ± 0.905	−16.939	< 0.001
Physical neglect	9.72 ± 2.970	8.56 ± 2.842	−13.217	< 0.001
Sexual abuse	5.72 ± 2.197	5.22 ± 1.066	−13.465	< 0.001

CSS, coping style scale for middle school students; MSSMHS, mental health scale for middle school students; CTQ-SF, childhood trauma questionnaire short form; NSSI, non-suicidal self-injury.

NSSI was selected as the dependent variable, with potential influencing factors acting as independent variables in the logistic regression analysis. Using the Forward: Logistic Regression method, we found that factors such as gender, interpersonal sensitivity, depression, coping style, and emotional abuse were linked to the occurrence of NSSI. The occurrence of NSSI in middle school students from families with dysfunctional parents was 1.322 times higher than those from families with harmonious relationships (OR = 1.322, 95% CI: 1.109–1.577). Students engaging in extracurricular activities exhibited less NSSI behavior (OR = 0.815, 95% CI: 0.710–0.935). The likelihood of NSSI occurrence was 0.271 times lower in students living with parents compared to those without (OR = 0.729, 95% CI: 0.624–0.851), signifying that living with parents can act as a protective factor against NSSI. Problem-oriented coping styles served as protective factors for NSSI, while emotion-oriented coping styles posed as risk factors (Table 3).

**Table 3:** Binary logistic regression analysis results of NSSI.

**Regression factor**	**Assignment**	***B***	**Exp (*B*)**	**OR (95% CI)**	***P*-value**
Gender	Girls = 0			1	
	Boys = 1	−0.441	0.070	0.643 (0.560 ~ 0.739)	< 0.001
Parental education	Both parents have lower education levels than high school = 0			1	
	Father or mother has a lower education level than high school = 1	0.048	0.076	1.049 (0.904 ~ 1.218)	0.528
	Both parents have higher education levels than high school = 2	0.250	0.093	1.285 (1.070 ~ 1.543)	0.007
Live with parents	Yes = 0			1	
	No = 1	−0.317	0.079	0.729 (0.624 ~ 0.851)	< 0.001
Parents often argue	No = 0			1	
	Yes = 1	0.279	0.090	1.322 (1.109 ~ 1.577)	0.002
Enjoy participating in extracurricular activities	No = 0			1	
	Yes = 1	−0.205	0.070	0.815 (0.710 ~ 0.935)	0.004
Emotional abuse		0.128	0.016	1.137 (1.101 ~ 1.174)	< 0.001
Obsessive-compulsive symptoms		−0.043	0.014	0.958 (0.933 ~ 0.984)	0.002
Hostility		0.071	0.012	1.074 (1.050 ~ 1.098)	< 0.001
Interpersonal sensitivity		0.044	0.013	1.045 (1.018 ~ 1.073)	0.001
Depression		0.132	0.013	1.141 (1.112 ~ 1.172)	< 0.001
Problem-oriented coping styles		−0.035	0.003	0.966 (0.959 ~ 0.972)	< 0.001
Emotion-oriented coping styles		0.023	0.004	1.023 (1.014 ~ 1.032)	< 0.001

CSS, coping style scale for middle school students; MSSMHS, mental health scale for middle school students; CTQ-SF, childhood trauma questionnaire short form; NSSI, non-suicidal self-injury.

## Discussion

Our study found that the detection rate of NSSI among middle school students in Hebei was 11.6% in the past year. The prevalence of NSSI in developing countries ranged from 11.5% to 33.8%, according to a retrospective study (25). In our study, the detection rate of NSSI was close to the lowest value, which may be influenced by the regional economic development level, related psychological education activities, and curriculum proliferation. In addition, cultural differences, including the importance of family and community, and views on NSSI, may also be the reasons for the different NSSI survey results in different countries (24). Due to cultural differences, in China, NSSI is often regarded as a “cowardly” behavior that cannot be understood by parents or classmates, which may affect their willingness to report NSSI and result in a lower detection rate than other studies. Some studies have used different scale assessment tools to evaluate NSSI behaviors, which may also lead to different examination rates (3). Of course, the two middle schools involved in this screening employ psychological teachers who regularly disseminate relevant psychological knowledge to students, alleviate students' negative emotions, and may consequently reduce the occurrence of adverse events.

This screening discovered that the majority of adolescents with NSSI were female, making up 58.3% of cases, a finding consistent with those of Andrews et al. (26). It could be that girls are more sensitive to negative emotions when confronted with stressors such as peer relationships, making them more likely to adopt negative coping styles. As a result, they may resort to NSSI as a means to alleviate their negative emotions (27). Some studies have also found that girls are highly correlated with “typically” feminine characteristics such as anxiety, depression, eating disorders, negative self-image, oversensitivity, which makes them more sensitive to emotional changes and more susceptible to negative emotions, thereby increasing the risk of developing NSSI (28).

This study discovered that adolescents who did not reside with their parents and had discordant family relationships were more prone to develop NSSI. A harmonious family environment plays a significant role in adolescent growth and development. Poor family interaction skills and a lack of positive emotional interchange amongst family members might create an unhealthy growth setting for adolescents. In strained family relationships, teenagers often resort to extreme measures such as self-harm to alleviate their inner negative emotions (29, 30). However, our study indicated that an increase in extracurricular activities might reduce such self-harming behaviors. This may be due to the release of beta-endorphins and a subsequent increase in endogenous opioid levels when participating in extracurricular activities, thereby reducing the inclination to self-harm (31, 32). Increasing outdoor activities for teenagers can help enhance interpersonal interaction, improve self-awareness and coping skills, thereby having a positive effect on their academic performance, health and behavior (33). Hence, enhancing students' extracurricular activities and balancing work and rest might decrease the incidence of NSSI in adolescents.

In our study, we found that emotional abuse was an independent risk factor for NSSI. This is in line with the findings of Xie et al. (7), who also reported that emotional abuse had the strongest association with NSSI. Childhood abuse can adversely impact an individual's attitudes and self-perceptions, as well as their outlook on life events and the future. These negative effects are often manifested in the form of exacerbated depressive symptoms and personality deficiencies (34). Moreover, childhood abuse may escalate the incidence of self-harming and suicidal behaviors—a harmful emotional coping mechanism that may persist into adolescence (7, 35).

This study revealed that the total score and dimension scores of the MSSMHS scale within the NSSI group were considerably higher than those in the non-NSSI group. Depression, hostility, subpar interpersonal relationships, and sensitivity can be potential risk factors for NSSI. Baetens et al. (36) discovered that positive interactions and increased peer contact may serve as protective factors against NSSI. On the other hand, poor interpersonal relationships, frequent disputes with classmates, and a lack of close friendships might heighten the risk of self-injury (37). A depressive mood can foresee the incidence of NSSI, and a significant amount of empirical literature has demonstrated that mood disorders and NSSI often transpire together (38). Compared to adolescents without NSSI, those with NSSI showed increased levels of anxiety and depression severity (39).

Adolescent behavior patterns may be influenced by different coping styles. This study found significant differences in problem-oriented and emotion-oriented coping styles between adolescents in the NSSI group and those without NSSI. The NSSI group scored higher on emotion-oriented coping styles than the non-NSSI group, while scoring lower on problem-oriented coping styles. This aligns with the findings of Jin et al. When adolescents encounter negative life events, emotion-oriented coping styles can often incite NSSI, with such self-harming behaviors frequently resulting from failures in emotional regulation (16, 20, 40). OR for emotion-oriented coping styles is minimal, but we should consider the practical significance of this factor.

This study has certain limitations. Firstly, this study is a cross-sectional study and cannot accurately explain the complex causal relationship between the research factors and NSSI. In the future, it is considered to add longitudinal studies to explore this topic. Secondly, the CTQ used in this study assesses the past experiences of the subjects and may have a certain degree of recall bias. Thirdly, the research was limited to Dingzhou City. Future research needs to expand the scale of the investigation to increase the universality of the research results.

## Conclusion

To sum up, the detection rate of NSSI within the past year in the city of Hebei stands at 11.6%, with a higher rate among girls than boys. Childhood trauma, emotional abuse, interpersonal relationship problems, depression and emotion-oriented coping styles are risk factors for NSSI, while problem-oriented coping styles are protective factors. Students with NSSI are more likely to be exposed to interpersonal relationship and study pressure problems. Considering the recognized risk and protective factors for NSSI, the possibility of NSSI may be decreased through the promotion of a harmonious home environment, encouraging positive emotional interaction, and the facilitation of greater involvement in extracurricular activities. Carrying out targeted extracurricular activities or family counseling programs, regularly holding parent-child activities, establishing a supportive school and family environment, and forming stable and close teacher-student or parent-child relationships can help build teenagers' sense of belonging to school and family, thereby enhancing their mental health. In conclusion, adolescent NSSI should receive extensive attention from all. Early identification of related risk factors and timely intervention may reduce the risk of NSSI.

## Data Availability

The raw data supporting the conclusions of this article will be made available by the authors, without undue reservation.
